# Rheumatic involvement and bone scan features in Schnitzler syndrome: initial and follow-up data from a single-center cohort of 25 patients

**DOI:** 10.1186/s13075-020-02318-5

**Published:** 2020-11-18

**Authors:** Christelle Darrieutort-Laffite, Catherine Ansquer, Hélène Aubert, Françoise Kraeber-Bodéré, Agathe Masseau, Christian Agard, Mohamed Hamidou, Claire Bernier, Jean-Marie Berthelot, Benoit Le Goff, Sébastien Barbarot, Antoine Néel

**Affiliations:** 1 Department of Rheumatology, CHU Nantes, 1 place Alexis Ricordeau, 44000 Nantes, France; 2 Department of Nuclear Medicine, CHU Nantes, Nantes, France; 3 Department of Dermatology, CHU Nantes, Nantes, France; 4 Department of Internal Medicine Interne, CHU Nantes, Nantes, France

**Keywords:** Bone scan, Bone lesions, Interleukin 1 receptor antagonist, Schnitzler syndrome

## Abstract

**Objective:**

To report on the characteristics and long-term course of rheumatic manifestations in Schnitzler syndrome (SchS).

**Methods:**

A retrospective cohort study of patients with SchS followed between 2000 and 2020. Inclusion criteria included a diagnosis of SchS (Strasbourg criteria). All available bone scans were reviewed and scored according to the intensity and number of pathological sites. The scintigraphic score was compared with the clinical activity score, CRP level, and treatments.

**Results:**

Twenty-five patients were included. Median age at diagnosis was 68 years. Eighty patients (72%) had SchS-related rheumatic pain. Most patients had a long-standing isolated rash before constitutional and/or rheumatic symptoms appeared. The monoclonal component level was usually very low (IgMκ in 22/25). Rheumatic pain predominated around the knees. Bone scans revealed abnormal tracer uptake in 15/18 (85%). The scintigraphic score correlated with clinical activity (*r* = 0.4, *p* < 0.02) and CRP level (*r* = 0.47, *p* < 0.01). The scintigraphic score was lower in patients receiving corticosteroids or IL1Ra (interleukin 1 receptor antagonist) than in untreated patients (median scores:2, 0, and 13, respectively; *p* < 0.05). Two patients developed Waldenström macroglobulinemia. Of the 22 surviving patients, median age at follow-up was 76 years. IL1Ra was used in 13 patients, with dramatic efficacy on both symptoms and bone scan features.

**Conclusions:**

Rheumatic manifestations are very prevalent in SchS. However, bone pain can be misleading and contribute to misdiagnosis. Bone scan abnormalities are very prevalent and correlate with disease activity and treatments. IL1-Ra has a dramatic and durable efficacy but may not be required in every patient early on.

## Background

Schnitzler’s syndrome (SchS) is a rare adult-onset inflammatory disease first described in 1972 by Liliane Schnitzler, a French dermatologist [1, 2]. SchS is characterized by the association of urticarial rash, monoclonal gammopathy (overwhelmingly IgMκ), and a variable combination of constitutional symptoms (fever, fatigue, weight loss), bone pain, osteosclerosis, and/or elevated inflammatory markers (erythrocyte sedimentation rate (ESR) or C-reactive protein (CRP)). SchS frequently has a significant impact on patients’ quality of life. Furthermore, the monoclonal component may evolve toward an overt hematologic malignancy (mostly Waldenström macroglobulinemia). Rare cases of AA amyloidosis have also been reported. In the past decade, IL-1 blockade (mostly with anakinra, off-label) have proved remarkably effective. Unfortunately, it is only suspensive [3–5]. In the absence of a known marker for a positive diagnosis, the diagnosis is currently based on the analysis of patients’ clinico-biological presentation, skin biopsy, bone imaging, and the exclusion of differential diagnoses. Diagnostic criteria were proposed in 2001 by Lipsker et al., with an urticarial rash and IgM component as 2 mandatory criteria [6]. More recently, the same group proposed and validated the so-called Strasbourg criteria, distinguishing patients with definite and probable SchS [7].

Despite the fact that clinical and radiological rheumatic involvement appear to be very prevalent in SchS series [4, 6–10], a clear picture of its natural history, diagnostic value, and long-term course is lacking.

Various imaging abnormalities have been reported, mostly in case reports: focal osteosclerosis, hyperostosis, periosteal reaction, and increased long bone uptake on bone scans [11–14]. Niederhauser et al. found radiologic abnormalities, predominantly located around the knees and in the innominate bone, in 14/22 patients [15]. Other groups reported much lower sensitivity of standard radiography (10–30%) [7, 16]. Bone scans have been reported to be a useful diagnostic tool [14]. However, how their findings correlate with clinical manifestations is unclear and whether therapy impacts the diagnostic value remains unknown.

The main objectives of the present study were to report on the clinical and imaging characteristics, as well as the long-term course of rheumatic manifestations in SchS. Our secondary objectives were to describe bone scan findings, to analyze their correlations with disease activity, and to determine whether therapy affected their diagnostic value.

## Methods

### Patients and data collection

We performed a retrospective study of patients diagnosed with Schnitzler syndrome that satisfied the Strasbourg criteria and who had been monitored at our center (Nantes University Hospital) since 2000. Bone scintigraphy became part of the initial baseline assessment of every patient with suspected SchS at that time. Cases were recruited from three departments: internal medicine, dermatology, or rheumatology. The following characteristics were collected: gender, age at disease onset, date of first symptoms and date of diagnosis, rheumatic, cutaneous and constitutional manifestations, biological features (monoclonal component, CRP, hemoglobin, leucocyte and platelet counts), treatments, and outcomes. This study was conducted in accordance with the Helsinki declaration and French ethics laws. The study design was in compliance with reference methodology MR003 (retrospective study of anonymized data with ethics approval waiver).

Physician assessment of SchS clinical activity was recorded using a semi-quantitative scale for rashes, pain, fever, and weight loss (0, absent-rare/1, moderate/2, frequent-severe), as reported previously [4]. The clinical activity score (range 0–8) was the sum of the scores of the 4 items.

### Bone scintigraphy

All patients underwent a whole-body bone scan performed 3 h after intra-venous injection of 99mTc-HDP. An additional tomoscintigraphy coupled with a CT scan (SPECT/CT) of the sites involved (mainly centered on the pelvis and knees) was performed in 11 patients.

All bone scans were reviewed and scored by the same senior nuclear medicine physician (CA), who was unaware of the clinical activity and treatments received. The bone scan was considered positive if abnormal uptake was observed in the usual sites of the disease [12–15] and negative if no abnormal or suggestive features of the disease were present. All focally radiotraced increased uptake in long bones (femurs, tibias, fibula, humerus, radius and ulna) and pelvic bone was collected and scored according to their intensity (1, faint; 2, moderate; and 3, high uptake). The scintigraphic score was determined for each patient by adding the intensity scores of each abnormal site.

### Statistics

The median and range were computed to describe the patient characteristics: age at onset, symptom duration, follow-up duration, CRP, hemoglobin, leucocyte and platelet counts, and bone scintigraphy score. Statistical analyses were performed using GraphPad Prism 8 software. Scintigraphic scores were compared between treated and untreated patients with a Mann-Whitney test, and comparisons of clinical activity, CRP level, and scintigraphic score before and after treatments were performed with a Wilcoxon test for paired data. Correlations between clinical activity, CRP level, and scintigraphic score were determined with a Spearman test. All tests were two-sided, and a *p* value < 0.05 was considered statistically significant.

## Results

### Rheumatic symptoms and SchS diagnosis

**Fig. 1:**
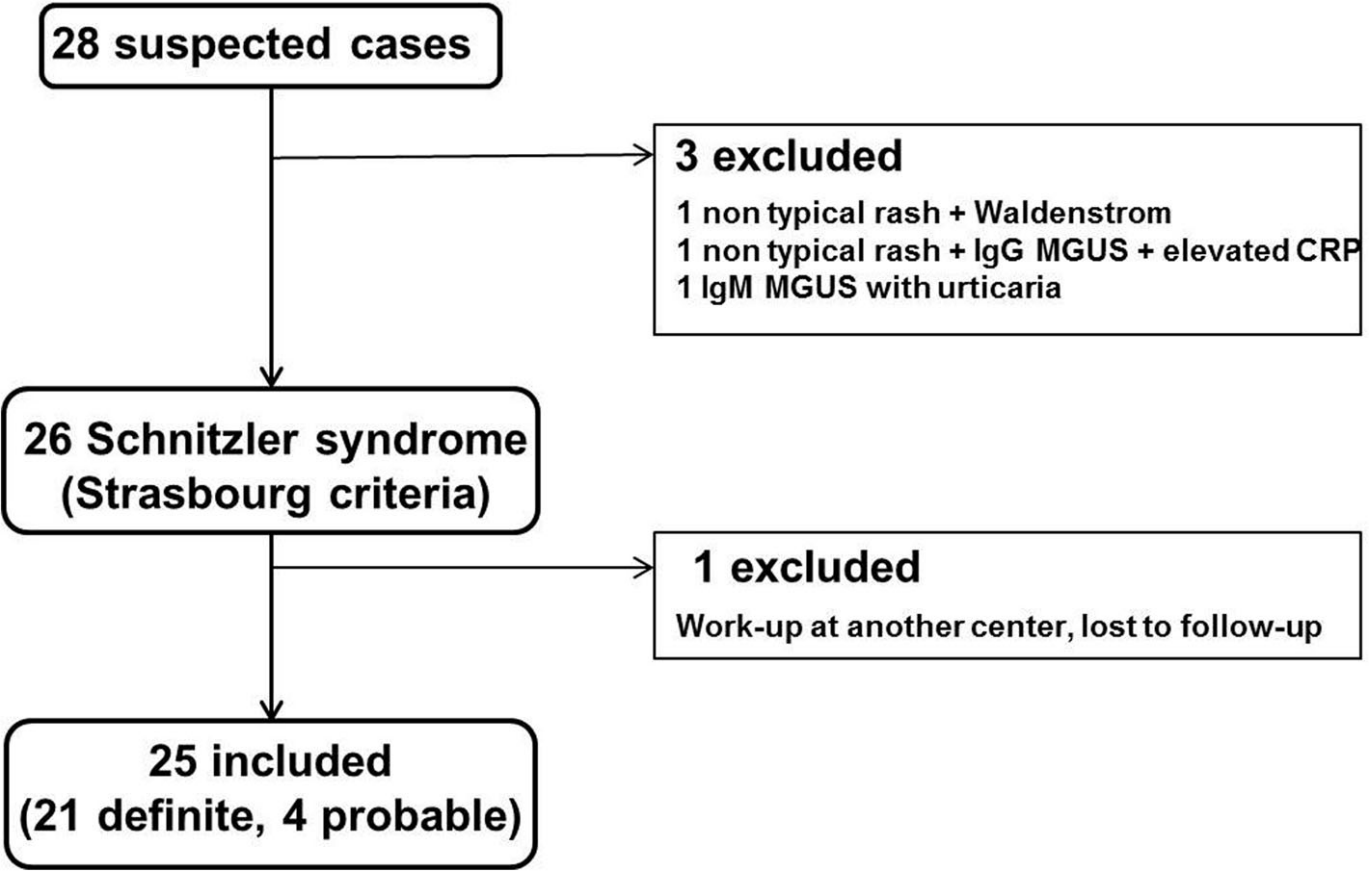
Study flow chart

**Fig. 2:**
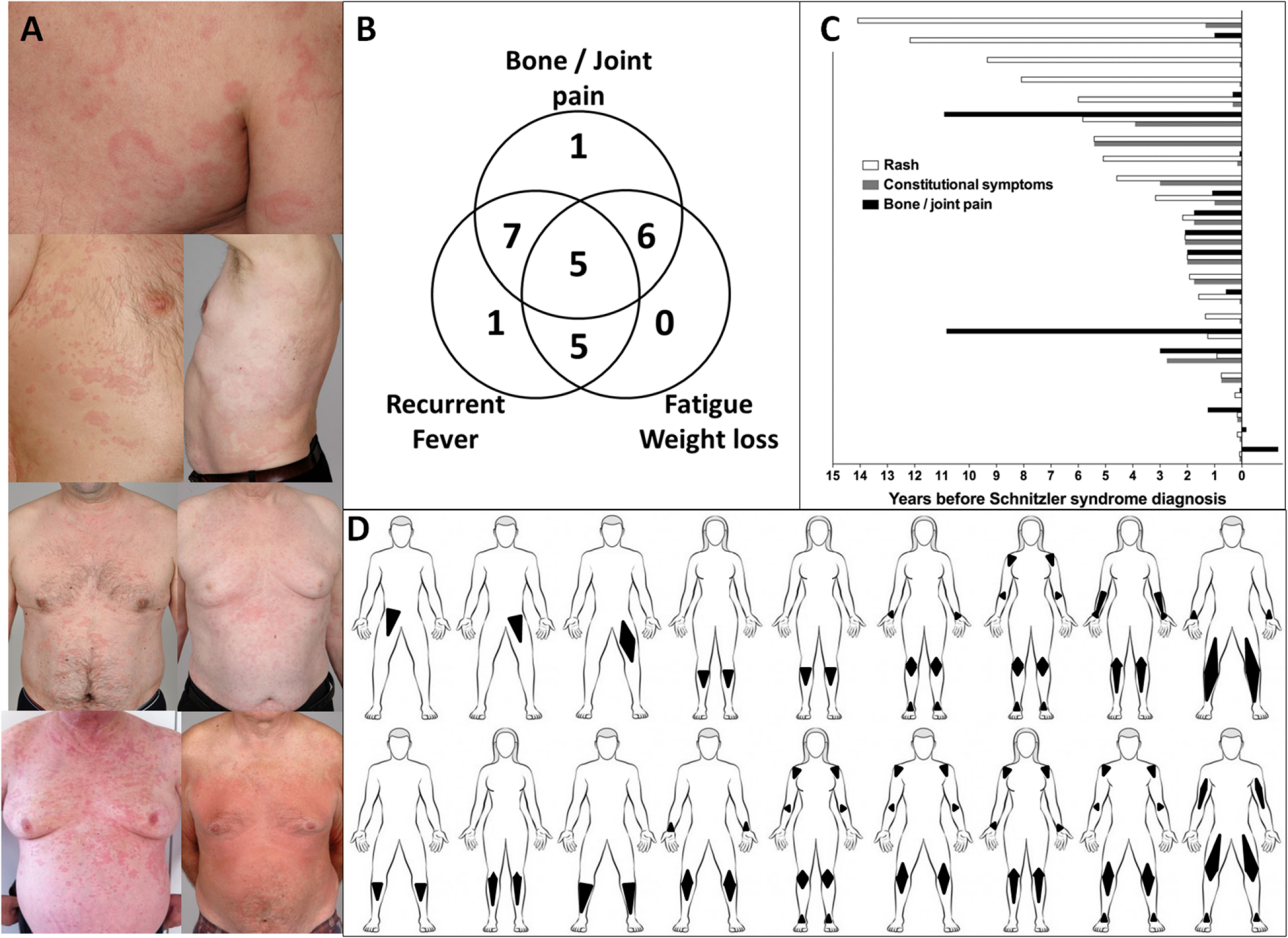
Cutaneous, rheumatic, and systemic clinical presentation of 25 patients with Schnitzler syndrome. **a** Representative example of typical rash in 7 patients. **b** Relationship between rheumatic and systemic symptoms. **c** History of symptom appearance prior to SchS diagnosis in 23 patients with detailed information. **d** Distribution of pain in 18 patients with clinical rheumatic involvement

Twenty-five patients were included (Fig. 1). By definition, all patients had an urticaria-like rash (Fig. 2a, b). Rheumatic pain was the second most common complaint (*n* = 18, 72%), along with fatigue (*n* = 18, 72%) and recurrent fever (*n* = 15, 60%). Median age at disease onset was 63 years (range 37–79). Most patients (*n* = 21/25, 83%) had a long-lasting, recurrent, or chronic rash frequently labeled as chronic spontaneous urticaria, before other symptoms of the disease developed. In 4 other cases, patients had not sought medical attention for their rash or had not noticed it. As depicted in Fig. 2c, patients’ histories were heterogeneous: some were pauci-symptomatic for years, whereas others appeared to have developed a rash with debilitating systemic and/or rheumatic symptoms over a few months. All patients had a monoclonal component, usually found at very low levels and only detected using immunofixation: its level was > 5 g/L in only 6 cases (25%) and too low to measure in 13 (42%). Only 3 patients had a non-IgMκ monoclonal component (Supplementary Table 1). All patients had an elevated CRP on more than 1 occasion. Most patients had marked systemic inflammation at time of diagnosis (Table 1), with anemia in 14 (56%) and thrombocytosis in 9 (36%). Of note, patients with intermittent symptoms could have no evidence of inflammation if evaluated at a period of low disease activity.

**Table 1:** Patients’ characteristics (*n* = 25)

Patients' characteristics	N (%) or median (IQR)
Male/Female	16/9
Age at disease onset (years)	63 (54–72)
Diag delay (months)	25 (13–67)
Rash	25 (100)
Monoclonal component	24 (100)
IgMκ	19 (76)
IgMκ+Mλ	2 (8)
IgMκ+Gκ	1 (4)
IgMλ	1 (4)
IgGλ	2 (8)
Rheumatic	21 (88)
Joint/bone pain	18 (75)
Abnormal imaging	19 (79)
Fatigue	18 (75)
Fever	15 (63)
Weight loss	14 (58)
Lymphadenopathy	7 (29)
CRP (mg/L)	80 (50–134)
Hemoglobin (g/dL)	11,4 (3, 8–13)
Platelet (G/L)	375 (294–468)
Leucocytes (G/L)	10,6 (1, 8–14)

Values indicate median (IQR) or *n* (%)

Pain affected the lower limbs in all patients and predominated around the knees (Fig. 2d). Pain was bilateral in 22 cases and unilateral in 3 cases (2 had isolated unilateral iliac bone involvement). Patients reported that the pain was exacerbated upon physical activities in 10 cases and at night in 11. None had morning stiffness or arthritis. As for treatment, 13/18 patients (72%) reported regular use of NSAIDs and/or painkillers. Data regarding conventional pelvic and long bone X-rays were available for 16 patients. Osteosclerotic lesions were observed in only 4 patients (25%) (Supplementary Figure 1): in the pelvis in 3 and in the femur in 2.

Expectedly, misdiagnosis was frequent until the whole clinico-biological picture led to suspicion of SchS. Patients’ pain had been attributed to osteoarthritis, spinal stenosis, venous insufficiency, rheumatic polymyalgia, peripheral neuropathy, or myalgia (suspected muscle vasculitis). Of note, 1 or more symptomatic rheumatic comorbidities was present in 7 patients, consistent with patients’ age (chondrocalcinosis in 1, hip or knee osteoarthritis in 2, lumbar spine stenosis in 2, painful sensory neuropathy in 1, wrist algoneurodystrophy in 1, shoulder calcific tendinopathy in 1, gluteus medius tendinopathy in 1). Once full-blown SchS had developed, the 3 most frequently suspected diagnoses were solid malignancy, lymphoma, and systemic vasculitis. All patients had had bone marrow examinations and 6 had had lymph node biopsies, which demonstrated reactive lymphadenitis. A skin biopsy was performed in 21 cases. An interstitial and/or perivascular dermal neutrophilic infiltrate was described in 10 cases. The most frequent clinical misdiagnoses were urticarial or unclassified small vessel vasculitis and temporal artery biopsy negative giant cell arteritis. No patients were initially misdiagnosed with cryopyrin-associated periodic syndrome or adult-onset Still disease. Median disease and extra-cutaneous symptom duration prior to diagnosis was 31 and 9 months, respectively.

### Bone scan features

All patients had had at least one bone scan, which was considered positive in 17 cases (68%). However, 4 out of 8 patients (50%) with a negative bone scan received corticosteroids or Interleukin 1 receptor antagonist (IL1Ra) (at the time of their first examination. Of the 4 untreated patients with a negative bone scan, 3 had no rheumatic symptoms.

**Fig. 3:**
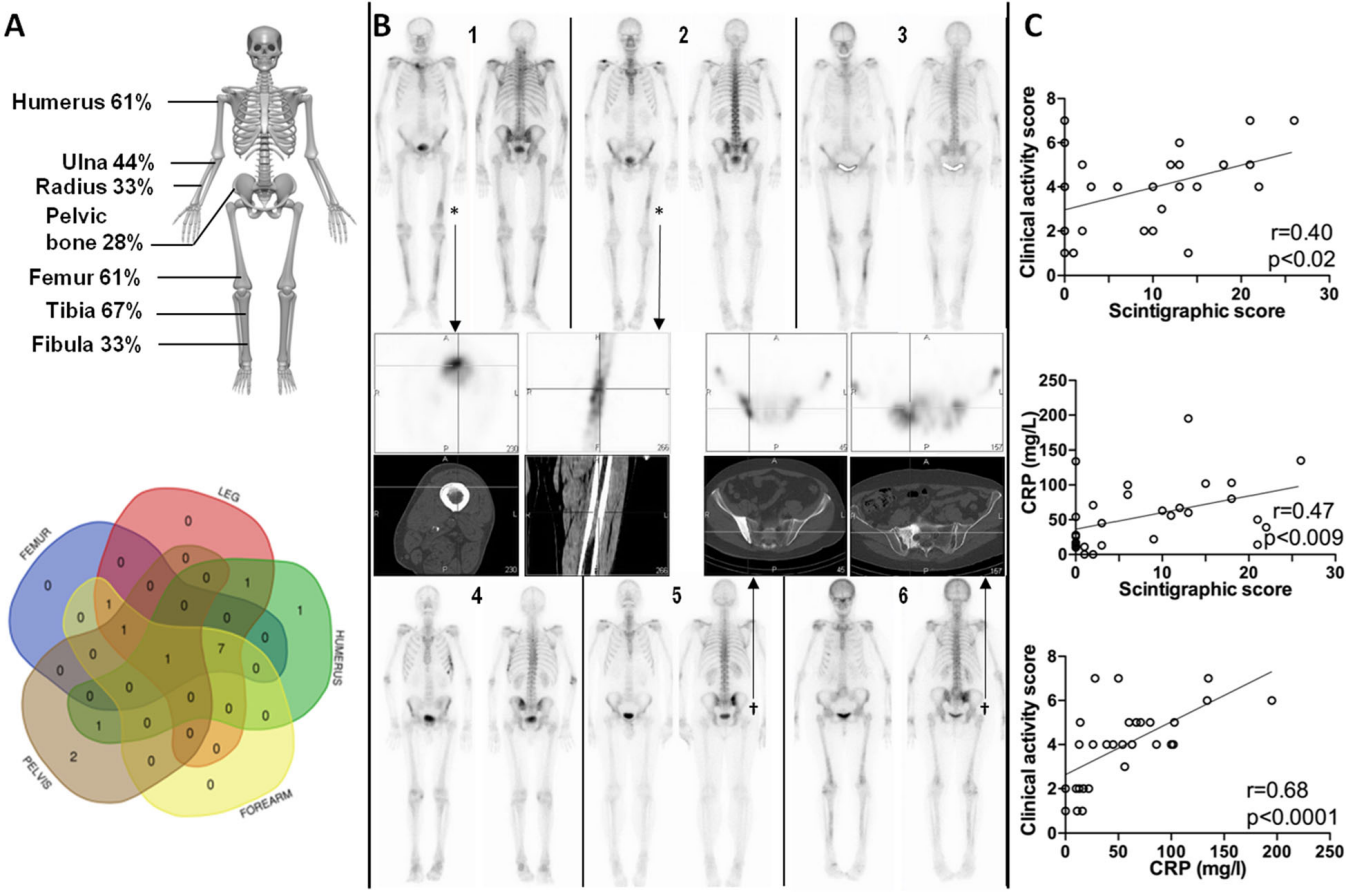
Bone scan findings in 18 patients. **a** Distribution of bone lesions [6]. **b** Representative example of long bones (patients 1–3 and 6) and/or pelvic bone involvement (patients 4–6) and selected cases with femoral (*) or pelvic (†) osteosclerosis on SPECT-CT. **c** Correlations between disease activity and bone scan features for patients without anti-IL1 treatment (Spearman)

In order to get a clear picture of the value of the bone scan, we decided to focus on 18 patients in whom a bone scan was performed at our center and was available for review. Of the 18 patients, 14 (78%) underwent a second exam, 5/18 (28%) a third and 2/18 (11%) a fourth. Overall, we thus analyzed 39 bone scans. At the time of their first bone scan, 13 patients were untreated, 5 were on steroids (median dose 9.5 mg/day), and none had received IL1Ra. We observed increased focal tracer uptake linked to SchS in 15/18 patients (83%) with a median number of bone lesions in 9 [range 1–12] and a median scintigraphic score in 13 [range 2–26]. Of the 13 patients assessed before treatment (Supplementary Table 2), bone lesions were observed in 11 cases (sensitivity of 85%). Increased tracer uptake was mainly located in long bones, as reported in Fig. 3a, b. Most patients had bilateral asymmetrical lesions in the femur and tibia, with/without upper limb involvement. Only 3 patients had a unique lesion which was, interestingly, located in the pelvis in 2 out of 3. Representative bone abnormalities are shown in Fig. 3b.

Of the 15 positive scintigraphies, 11 were obtained in untreated patients and 4 in patients on steroids (median dose 8.5 mg/day). Twelve out of the 15 patients with a positive bone scan (80%) suffered bone pain and 3 did not. Of the 3 patients with a negative bone scan, 2 suffered from bone pain, including 1 treated with corticosteroids. Thus, only a single patient had neither pain nor bone lesions. The relationships between pain, bone lesions, and treatment are reported in Supplementary Table 3. Disease duration was similar between patients with or without bone lesions at diagnosis.

In order to determine whether bone scan abnormalities correlated with clinical and/or biological disease activity, we focused on bone scans performed without anti-IL1 (interleukin 1) treatment (*n* = 25). We found a significant correlation between scintigraphic score and both clinical activity score and CRP level (Fig. 3c).

Overall, these data show that scintigraphic bone involvement is highly prevalent in SchS, with a significant yet incomplete overlap between clinical and imaging assessment. Furthermore, bone scan abnormalities correlate with disease activity.

### Impact of treatments on bone scan features

**Fig. 4:**
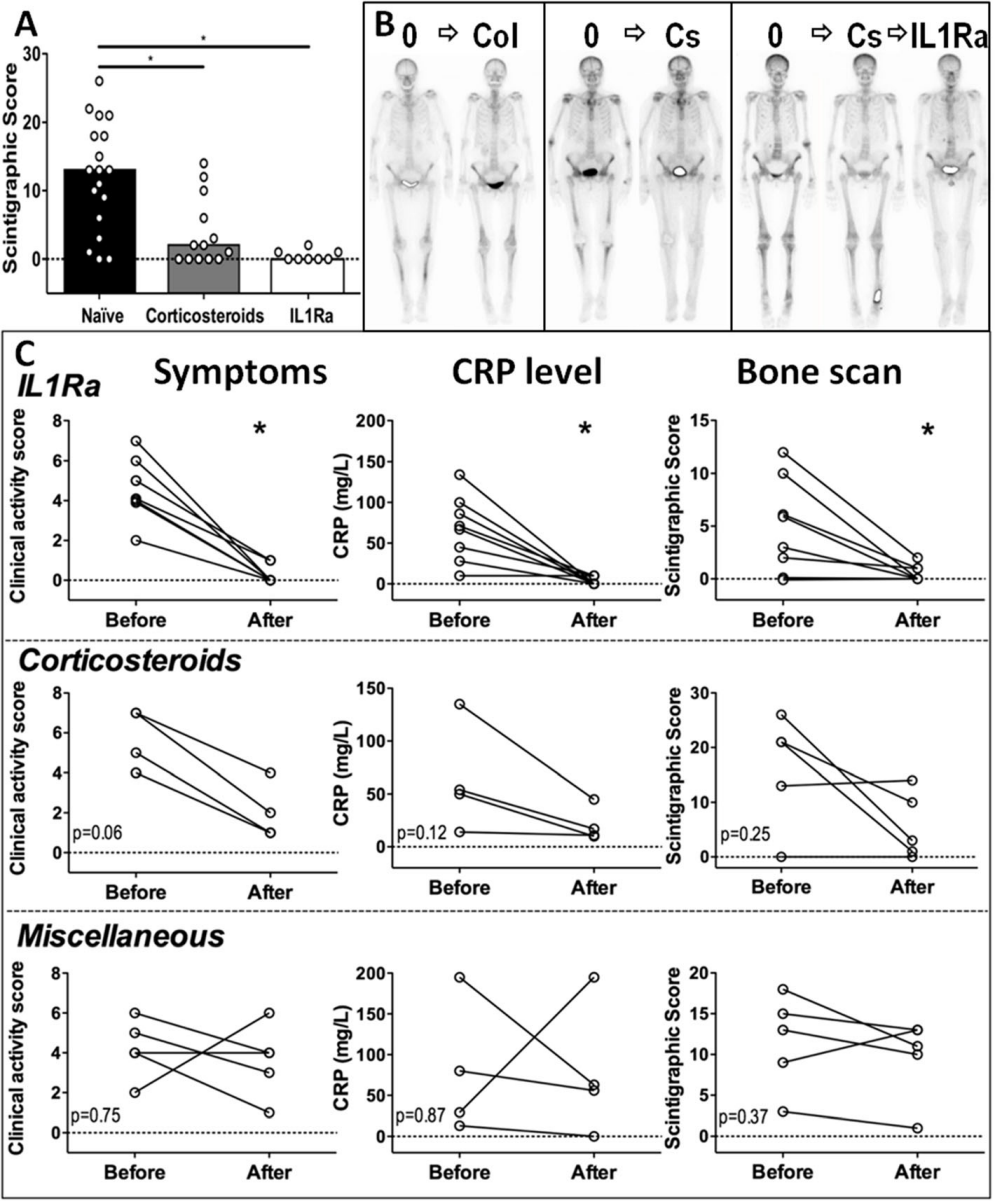
Schnitzler syndrome treatment impact on bone scan findings. **a** Unpaired comparison of bone scan scores according to patients’ treatments. **b** Representative example of the impact of therapy on bone scan images (0, none; Col, colchicine; Cs, corticosteroids). **c** Clinical score, CRP level, and scintigraphic score changes according to therapy

Patients with SchS frequently experience a significant therapeutic delay. Some of them are treated with steroids with an alternative diagnosis when the correct diagnosis is eventually considered. Having shown that bone scans have good sensitivity, we wanted to determine whether it could be diminished in patients treated with corticosteroids. We thus analyzed scintigraphic scores in relation to patients’ therapeutic regimen. We observed a higher scintigraphic score in patients who received neither steroids nor IL1-Ra compared to patients on steroids or those receiving IL1-Ra (median scores 13, 2, and 0, respectively) (Fig. 4a, b). These data suggest that corticosteroids can reduce ^99m^Tc-HDP uptake, thereby resulting in false-negative results.

To further document the impact of treatment on bone scans, we analyzed paired imaging, in 14 patients after starting corticosteroids (*n* = 5), or IL1Ra (*n* = 9), or left without specific treatment (*n* = 5). The latter group was either untreated or received anti-H1, painkillers, on demand short NSAID courses, and/or colchicine. Of the 8 patients who started on IL1Ra, 6 had failed corticosteroids (Fig. 4c). Corticosteroids led to a partial improvement in clinical activity, CRP level, and bone scan scores. Expectedly, IL1Ra had dramatic clinical and biological efficacy, with a major impact on bone scan abnormalities. Three out of 8 patients (37%) had a complete resolution of their scintigraphic lesions when taking IL1Ra.

### Long-term patient outcomes

Follow-up information was available for 24 patients. At last follow-up, median disease duration was 13 years (IQR 9.8–15; range 0–22). Median duration of follow-up since the diagnosis of SchS was 7.5 years (IQR 4–12, range 1–17). The most frequently used treatments were corticosteroids (*n* = 17), colchicine (*n* = 15), and IL1Ra (*n* = 13). Efficacy data are reported in Supplementary Table 4.

When last seen, all 13 patients treated with IL1Ra were still receiving the drug (mean treatment duration: 6.8 years [IQR 3.3–8.9, range 0.1–17]). A single patient experienced occasional rash despite daily injections and normal CRP levels. Only 2 patients were able to taper IL1Ra to alternate day injections. Two patients were still on low dose steroids due to residual lower limb pain despite complete remission of the rash and consistently normal CRP levels (1 had hip osteoarthritis, 1 spinal stenosis).

**Fig. 5:**
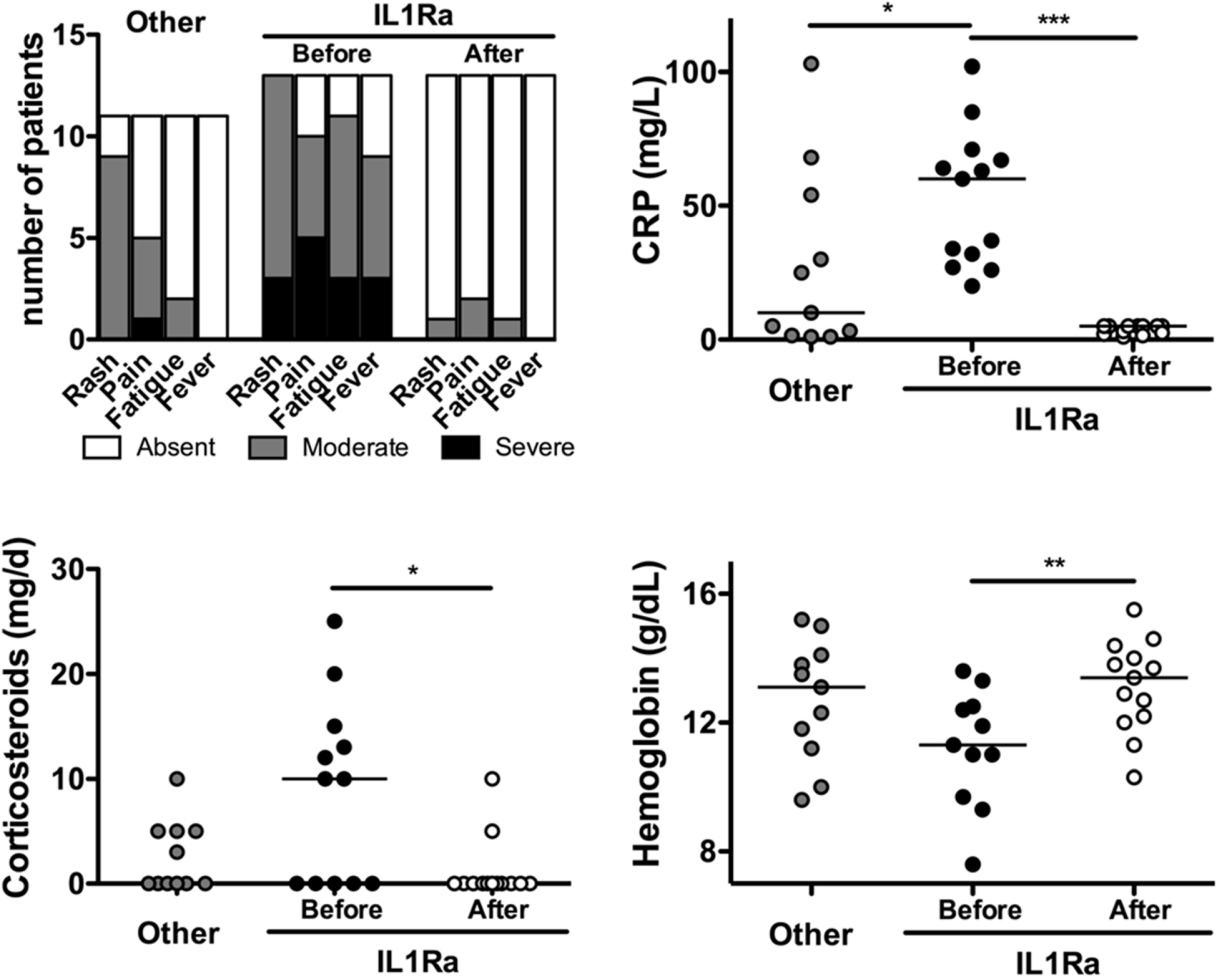
Long-term follow-up of 24 patients: comparison of clinico-biological data before IL1Ra initiation to last follow-up data of IL1Ra-treated and IL1Ra-naïve (“other”) patients. *p < 0.05, **p < 0.01, ***p < 0.001

Of the 11 IL1Ra-naïve patients, 2 patients did not require treatment, 1 became asymptomatic on mycophenolate mofetil (for auto-immune hepatitis), 2 were on low-dose steroid monotherapy (< 5 mg/day), and 6 were on colchicine as a monotherapy (*n* = 1), or in combination with on demand NSAIDs (*n* = 2) or low dose steroids (*n* = 3). Clinical and biological information at last follow-up is shown in Fig. 5.

Only 2 patients evolved toward a hematologic malignancy (Waldenström macroglobulinemia): 1 patient was diagnosed with Schnitzler syndrome several years after being treated for Waldenström macroglobulinemia, while he had suffered isolated urticaria for more than 10 years. Another patient developed Waldenström macroglobulinemia 12 years after an expedite diagnosis of SchS. He never required chemotherapy until his death from an unrelated cause (stroke). None of our patients developed amyloidosis. At the time of data acquisition (late 2019), 3 patients had died (sepsis in 2, stroke in 1, ages 70, 75, and 90 years), 5 were lost to follow-up, and 16 patients were in active follow-up. Of the living patients, median age at last follow-up was 76 years (IQR 67–80, range 52–88).

## Discussion

SchS is a rare, poorly understood inflammatory disease characterized by the association of a chronic relapsing urticarial rash with a typically IgMκ monoclonal component and a variable combination of constitutional symptoms (fever, weight loss, fatigue), rheumatic manifestations (pain, osteosclerosis), and recurrent or chronic biological inflammation. SchS can severely affect patients’ quality of life [17], which can nowadays be transformed by the off-label use of IL1Ra [4, 17]. Despite their high prevalence and significant burden, the rheumatic manifestations of SchS have not been given much attention. The purpose of our study was to report on the clinical and imaging features of this aspect of the disease.

In 2013, a large scale study performed at the Mayo Clinic elegantly demonstrated that SchS was an underdiagnosed entity [8]. Between 1972 and 2010, only 16 patients were diagnosed with SchS out of 4103 patients with an IgM MGUS. In the same period, 46 cases went unrecognized, despite frequently suffering fever (54%) and/or bone pain (78%). In our center, we observed that SchS was not that exceptional a disease, as reflected by the number of cases we encountered over only 2 decades, making this the largest European single-center series published.

Expectedly, rheumatic pain was highly prevalent in our patients, consistent with previous series in which it was reported in 70–80% of cases. Most of our patients had ill-defined lower limb and/or widespread pain. Pain exacerbation at night was not rare. Morning stiffness and arthritis were never reported. Older reports were mostly based on the analysis of previously published cases [6, 9] where the origin of patients’ pain was unclear. Our clinical and imaging findings strongly suggest that despite being frequently interpreted as arthralgia and sometimes myalgia, SchS-related rheumatic pain is the result of bone involvement.

All our patients had urticarial rash at presentation. Their rash was frequently misdiagnosed as chronic spontaneous urticaria, despite [18, 19]. As emphasized in the literature, SchS rash, which is a neutrophilic urticarial dermatosis, in most cases mainly affected the trunk and limbs, sparing the palms, soles, and face [20]. It could be papular but also macular, red but occasionally pale pink, and was rarely itchy. Despite this key symptom, our patients suffered a significant diagnostic delay, which in our view was the result of 4 factors. First, the frequently significant lag time between rash appearance and extra-cutaneous manifestations tended to prevent clinicians from suspecting a unicist diagnosis. Second, the patients’ older age and rheumatic comorbidities altered the clinicians’ approach to rheumatic symptoms. Third, conventional X-rays rarely demonstrated osteosclerosis. And finally, the monoclonal component was usually present at low levels and only detected by immunofixation, meaning there would have to have been suspicion of the diagnosis already.

In recent years, several groups have pointed to bone scans as an appealing tool for the documentation of bone lesions in SchS [15, 16, 21], but their sensitivity had not been well determined. In a study of bone and angiogenesis blood markers reported by Terpos et al., all 13 patients had a positive bone scan [16]. In contrast, in the study that validated the Strasbourg criteria, bone scan sensitivity appeared to be 60% [7]. Here, we demonstrate the value of systematic whole-body bone scan imaging in patients with SchS. We found that its sensitivity was 85%. Bone scans demonstrated long bone lesions—predominantly around the knees—and/or pelvic bone involvement (mostly the ilium). Bone scans correlated well with clinical manifestations (pain location and disease activity) but could also demonstrate bone involvement in patients without bone or joint pain. In our practice, we found that in patients with urticarial rash and elevated acute-phase reactants plus pain and/or constitutional symptoms (fever, weight loss, fatigue), identifying an IgMκ from immunofixation and typical bone involvement on scintigraphy are the 2 pillars of the diagnosis of SchS.

As discussed by Gusdorf et al., the impact of therapy on bone scans had never been reported previously [7]. We demonstrate that corticosteroids can reduce bone scan abnormalities, which may explain discrepancies in the data in the literature. Clinicians should be aware of this possibility when considering the diagnosis of SchS in patients who have already been treated with steroids. As for patient follow-up, we found that bone scan findings correlated with clinical and biological manifestations and thus provided no additional information to guide therapy, which targets patient’s symptoms and quality of life.

SchS is a two-faced disease, which alters quality of life in the short term but can also pose a vital threat to patients in the long term [9, 22]. The 2 complications of the disease are MGUS progression toward an overt lymphoproliferative disease (mostly Waldenström macroglobulinemia) and AA amyloidosis resulting from chronic inflammation. These complications appear rather infrequently, both in our experience and in recently published series. The 10-year risk of developing WM has been estimated at 15% by de Koening et al. [9]. More recently, Jain et al. reported that 8% of the Mayo clinic cases evolved toward a lymphoproliferative disease, which is consistent with our observation [8]. As for AA amyloidosis, it has been reported in 2% of published cases according to Rowczenio et al. [17] Consistently, with this estimate, in our previous multicenter study of 42 cases, only 1 developed AA amyloidosis, which was present at diagnosis [4]. Little scientific evidence can guide the management of SchS. An international expert panel proposed therapeutic recommendations which discussed the value of colchicine, NSAIDs, Peflacine, and, above all, IL1Ra [21]. Canakinumab has also been proven effective [23]. In a recent UK series, all patients received IL1Ra [17]. At our center, IL1Ra is only used after failure of symptomatic treatments and variable combinations of colchicine, NSAIDs, and/or low-dose corticosteroids (if considered safe), also considering patients’ comorbidities and preferences. Our results emphasize the value of colchicine in patients with the least severe forms of the disease. However, the majority of cases will probably require an IL1 blockade in the long term. The dramatic efficacy of an IL1 blockade has shed new light on the pathogenesis of SchS, but almost half a century after its first description, the relationships between the monoclonal-component secreting small B cell clones, IL1 overproduction, myeloid cells (monocytes, mast cells), and target organs (bone, skin) are yet to be fully understood [16, 24–30].

The main limitations of this study are related to the retrospective design with missing or imprecise data. However, given the rarity of the disease, it would take many years to complete a prospective study.

## Conclusions

We report on the analysis of a cohort of 25 SchS patients, demonstrating the burden of the rheumatic manifestations of this disease, its characteristics, diagnostic pitfalls, and the high diagnostic value of bone scans, even in asymptomatic patients. Bone scan should be routinely part of the diagnostic work-up of patients with suspected Schnitzler syndrome. Clinicians should be aware that corticosteroids can diminish scintigraphic abnormalities. When facing a patient with urticarial rash, rheumatic pain, and/or constitutional symptoms, a high level of suspicion is needed given the frequently debilitating consequences of the disease and the dramatic efficacy of IL1Ra.

## Supplementary information

**Additional file 1 : Figure S1.** Conventional x-rays showing osteosclerotic lesions of the femur (A,B) and bone densification of the iliac bone (C,D). **Table S1.** Characteristics and outcome of 3 non IgMκ cases. **Table S2.** Characteristics of 13 patients assessed with bone scan while untreated. **Table S3.** Relationship between Bone scan features, pain, and treatment. **Table S4.** Treatments.

## Data Availability

The datasets used and/or analyzed during the current study are available from the corresponding author on reasonable request.

## Abbreviations

SchS: Schnitzler syndrome
ESR: Erythrocyte sedimentation rate
CRP: C-reactive protein
SPECT/CT: Tomoscintigraphy coupled with a CT scan
IL1RA: Interleukin 1 receptor antagonist
IL1: Interleukin 1
