# Supplementary material for: Rheumatic involvement and bone scan features in Schnitzler syndrome: initial and follow-up data from a single-center cohort of 25 patients
Source: Arthritis Res Ther. 2020 Nov 18;22:272. doi: 10.1186/s13075-020-02318-5 (PMC7677784; doi:10.1186/s13075-020-02318-5)
Supplement: Supplementary file 1 — Additional file 1 : Figure S1. Conventional x-rays showing osteosclerotic lesions of the femur (A,B) and bone densification of the iliac bone (C,D). Table S1. Characteristics and outcome of 3 non IgMκ cases. Table S2. Characteristics of 13 patients assessed with bone scan while untreated. Table S3. Relationship between Bone scan features, pain, and treatment. Table S4. Treatments. [file 13075_2020_2318_MOESM1_ESM.pdf]

SUPPLEMENTARY DATA

Supplementary figure 1. Conventional x-rays showing osteosclerotic lesions of the femur (A,B) and bone densification of the iliac bone (C,D).

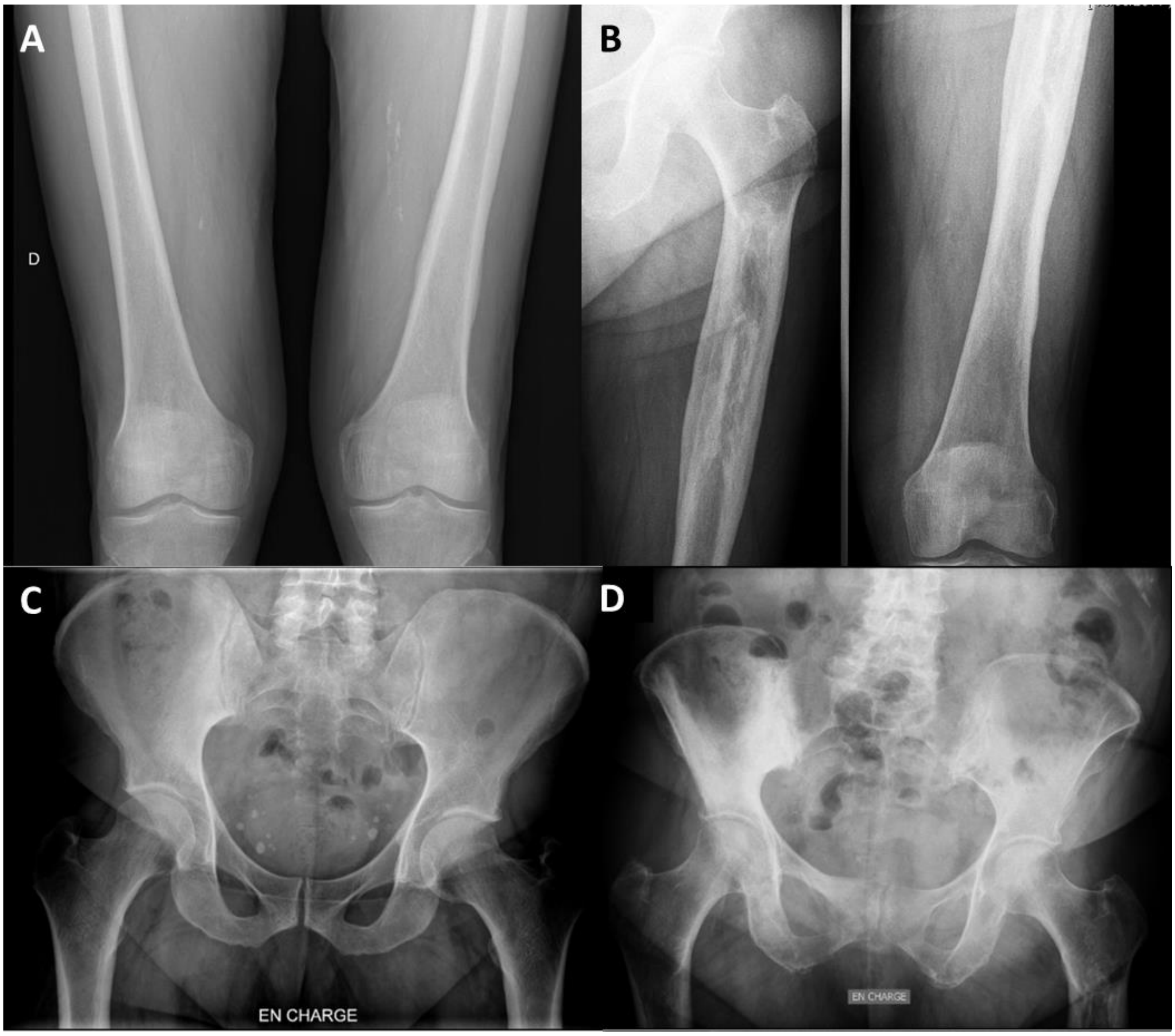

Supplementary Tables

Supplementary Table 1: Characteristics and outcome of 3 non IgMκ cases

Sex	Age	Department	Monoclonal component	Rash	Skin biopsy	Recurrent Fever	CRP level (mg/L)	Joint/bone pain	Bone scan	Strasbourg criteria	Response to IL1Ra	Follow-up
F	62	Internal Medicine	IgMλ	Typical	Neutrophilic dermal infiltrate	Yes	139	Yes (knee/tibia)	Typical	Definite	NA	2y (alive)<br>PR under low dose steroids and colchicine
M	51	Dermatolog y	IgGλ	Typical	Neutrophilic dermal infiltrate	Yes	69	Yes<br>(knee/tibia)	Negative under IL1Ra	Definite	CR	17y (alive)<br>CR under IL1Ra (13 years)
F	79	Dermatolog y	IgGλ	Typical	Neutrophilic dermal infiltrate	Yes	17	No	Negative under steroïds	Probable	NA	6y (deceased)<br>PR under low dose steroids

CR : complete remission, PR : partial remission

Supplementary Table 2 : Characteristics of 13 patients assessed with bone scan while untreated.

M/F	6/7	
Age at disease onset (y)	69	(60-71)
Diag delay (mo)	19	(6-26)
Rash	25	(100)
Monoclonal component	24	(100)
IgMκ	12	(92)
IgMκ+Mλ	1	(8)
Joint/Bone Pain	10	(77)
Fatigue	10	(77)
Fever	8	(61)
Weight loss	7	(54)
Lymphadenopathy	4	(31)
CRP (mg/L)	101	(55-134)
Hemoglobin (g/dL)	11,5	(9,8-13)
Platelet (G/L)	378	(343-468)
Leucocytes (G/L)	11,7	(10,3-13,2)
Treatment during follow-up		
Colchicine	8	(61)
CR / PR / NR	0/7/1	
Corticosteroids	10	(77)
CR / PR / NR	6/4/0	
IL1Ra	8	(61)
CR / PR / NR	8/0/0	
Other*	3	(23)

Values indicate median (IQR) or n (%).
CR : complete response, PR : partial response, NR: non response
* Biphosphonate infusions, Mycophenolate, Peflacine (1 each)

Supplementary Table 3: Relationship between Bone scan features, pain, and treatment

	Bone/Joint Pain (n=14)		No Pain (n=4)	
	Untreated	Treated*	Untreated	Treated*
positive bone scan (n=15)	9	3	2	1
negative bone scan (n=3)	1	1	1	0

*corticosteroids

Supplementary Table 4: Treatments

	n	response		
		CR	PR	NR
Corticosteroids	17	10	6	1
Colchicine	15	0	12	3
Anakinra	13	13	0	0
Peflacine	2	0	1	1
Chloraminophene	2	0	0	2
Methotrexate	2	0	0	2
Mycophenolate	1	1	0	0
Rituximab-Fludarabine	1	0	1	0

CR: complete response, PR: partial response,
NR: non response

Complete Response : complete disappearance of rash, SchS-related pain, systemic symptoms and a normal CRP level.

Partial Response : improvement of skin rash plus at least 1 systemic manifestation including fever, bone pain and fatigue, which allowed reduction of other treatments (Painkillers, NSAID, corticosteroids) plus decrease in CRP level.
